# SMRT Sequencing Reveals Candidate Genes and Pathways With Medicinal Value in *Cipangopaludina chinensis*

**DOI:** 10.3389/fgene.2022.881952

**Published:** 2022-06-16

**Authors:** Kangqi Zhou, Zhong Chen, Xuesong Du, Yin Huang, Junqi Qin, Luting Wen, Xianhui Pan, Yong Lin

**Affiliations:** Guangxi Key Laboratory for Aquatic Genetic Breeding and Healthy Aquaculture, Guangxi Academy of Fishery Sciences, Nanning, China

**Keywords:** *Cipangopaludina chinensis*, full-length transcriptome, SMRT sequencing, functional annotation, structure prediction

## Abstract

*Cipangopaludina chinensis* is an economically important aquatic snail with high medicinal value. However, molecular biology research on *C. chinensis* is limited by the lack of a reference genome, so the analysis of its transcripts is an important step to study the regulatory genes of various substances in *C. chinensis*. Herein, we conducted the first full-length transcriptome analysis of *C. chinensis* using PacBio single-molecule real-time (SMRT) sequencing technology. We identified a total of 26,312 unigenes with an average length of 2,572 bp, of which the largest number of zf-c2h2 transcription factor families (120,18.24%) were found, and also observed that the majority of the 8,058 SSRs contained 4-7 repeat units, which provided data for subsequent work on snail genetics Subsequently, 91.86% (24,169) of the genes were successfully annotated to the four major databases, while the highest homology was observed with *Pomacea canaliculata*. Functional annotation revealed that the majority of transcripts were enriched in metabolism, signal transduction and Immune-related pathways, and several candidate genes involved in drug metabolism and immune response were identified (e.g., CYP1A1, CYP2J, CYP2U1, GST, ,PIK3, PDE3A, PRKAG). This study lays a foundation for future molecular biology research and provides a reference for studying genes associated with the medicinal value of *C. chinensis*.

## 1 Introduction

*Cipangopaludina chinensis* is a common, large freshwater snail, that is, widely distributed in lakes, rice fields, ponds and other waters around the world. As a primary consumer, it plays an important role in energy transfer and water purification in the ecosystem. The meat and shell of the snail have edible and medicinal value, can be used as feed for poultry and aquatic animals, and are favored by consumers and farmers (Chen L. et al., 2019; Luo et al., 2021; Zhou et al., 2021). In recent years, with the rise of the Guangxi snail rice noodle industry in China, the demand for snails has increased, which has promoted the development of the snail industry from relying on natural fishing to artificial breeding (Qin., 2020). As a result, scholars are paying increasing attention to this snail farming model (He et al., 2018; Lei., 2020; Qiu., 2020; Jie., 2021; Wu et al., 2021). Some researchers have explored the potential of *C. chinensis* in medicine. In an alcohol-induced liver injury model, the significant antioxidant and hepatoprotective activity of *C. chinensis* polysaccharide (CCPS) was demonstrated (Jiang et al., 2013; Fan et al., 2014). And also found that CCPS can inhibit neovascularization of endothelial cells by mediating their “proliferation, migration and lumen formation” (Xiong et al., 2017; Xiong et al., 2020). Meanwhile, an *in vitro* model using a 2.2.15 cell line of human hepatocellular carcinoma cells (HepG2) revealed the significant anti-hepatitis B virus effect of CCPS (Liu et al., 2013). However, fewer studies have focused on the transcriptome level from the perspective of *C. chinensis* molecular biology and breeding so far.

Currently, single-molecule real-time (SMRT) long-read sequencing technology based on PacBio platform is the most reliable full-length cDNA molecular sequencing method, which can truly reflect the transcriptome information of the sequenced species and is conducive to the study of variable splicing, gene fusion, alleles and other mRNA structures, and is now widely used in the whole transcriptome analysis of plant and animal species (Korlach., 2013; Roberts et al., 2013; Lin et al., 2017; Ardui et al., 2018; Li et al., 2018; Chen S. et al., 2019; Yang et al., 2020). Here, based on the Sequel platform, we used PacBio’s SMRT to sequence the full-length transcriptome of *C. chinensis*. Then, we conducted a systematic analysis of all sequences, including functional annotation and identification of simple sequence repeats (SSRs), coding DNA sequences (CDSs), non-coding RNAs (ncRNAs), transcription factors (TFs), and alternative splicing (AS) events. We aimed to obtain a large amount of useful sequence information. This study obtained a full-length transcriptome of this snail for the first time, which will provide valuable data for subsequent gene function research, molecularly assisted breeding, and biomedical functions.

## 2 Materials and Methods

### 2.1 Sample Collection and RNA Extraction

A healthy individual of *C. chinensis* (weight 20.30 g, shell height 46.05 mm, shell width 33.27 mm) was collected from the rice snail breeding demonstration base in Ligao Village (23.37°N, 111.29°E), Liuzhou, Guangxi, China. Eight tissues were collected, namely the foot, intestine, hepatopancreas, gill, gonad, esophagus, blood, and lip. The samples were frozen with liquid nitrogen and stored at −80°C for later use. The trial was performed in accordance with the Institutional Animal Care and Use Committees of Guangxi Academy (CGA-00927).

The total RNA from these tissues was extracted separately, and equal amounts of RNA were mixed to construct a library after quality testing. Briefly, total RNA was extracted by grinding each tissue in TRIzol reagent (Life technologies, United States) on dry ice and processed following the manufacturer’s protocol. The integrity of the RNA was determined with an Agilent 2,100 Bioanalyzer and agarose gel electrophoresis. The purity and concentration of the RNA were determined with a Nanodrop micro-spectrophotometer (Thermo Fisher).

### 2.2 Library Construction and Single-Molecule Real-Time Sequencing

Referring to the methods of Huang et al., (2021). Briefly, mRNA was enriched using Oligo (dT) magnetic beads (PacBio biosciences, United States). The enriched mRNA (2 μg) was reverse transcribed into cDNA using a Clontech SMARTer PCR cDNA Synthesis Kit (Clontech, United States). PCR cycle optimization was used to determine the optimal amplification cycle number for the downstream large-scale PCR reactions. The optimized cycle number was then used to generate double-stranded cDNA. In addition, >5-kb size selection was performed using the BluePippin™ Size-Selection System and the resulting cDNA was mixed equally with non-size-selected cDNA. Large-scale PCR was performed for subsequent SMRTbell library construction. The cDNA samples were barcoded, then pooled and constructed as a “single” sample in the SMRTbell library, with different barcodes distinguishing the data from the different samples. The cDNAs were DNA damage repaired, end repaired, and ligated to sequencing adapters. The SMRTbell template was annealed to the sequencing primer and bound to polymerase, and sequenced on the PacBio Sequel II platform by Gene Denovo Biotechnology Co. (Guangzhou, China). A total of 57 Gb reads were generated from the SMRT library.

### 2.3 Data Processing

The raw sequencing reads of the cDNA libraries were analyzed using the unigene sequencing (Iso-Seq) pipeline supported by Pacific Biosciences (Gordon et al., 2015). First, high-quality circular consensus sequences (CCSs) were extracted from the subreads BAM file. The integrity of the transcripts was judged on the basis of whether the CCS reads contained 5′ primer, 3′ primer, and poly A structures. The sequences containing all three structures were called full-length sequences (FL reads). Subsequently, primers, barcodes, poly A tails, and concatemers of full passes were removed to obtain full-length non-chimeric (FLNC) reads. The FLNC reads were clustered to generate complete unigenes. Similar FLNC reads were clustered hierarchically using minimap2 to obtain a consistent sequence (unpolished consensus unigenes) (Li., 2018). The quiver algorithm was then used to further correct the consistent sequences. Two strategies were used to improve the accuracy of PacBio reads. First, we used non-full-length reads to polish the obtained cluster consensus Unigenes with the Quiver software to produce FL, polished, high-quality consensus sequences (accuracy of ≥99%). Second, Illumina short reads were obtained from the same samples with the LoRDEC to further correct low-quality isoforms (Salmela & Rival, 2014). On the basis of the results, both high-quality unigenes (prediction accuracy was ≥ 0.99) were combined for subsequent analysis.

### 2.4 Basic Annotation of Unigenes

To annotate them, the unigenes were BLAST analyzed against the NCBI non-redundant protein (Nr) database (https://www.ncbi.nlm.nih.gov), the Swiss-Prot protein database (https://www.expasy.ch/sprot), the Kyoto Encyclopedia of Genes and Genomes (KEGG) database (Kanehisa & Goto, 2000), and the COG/KOG database (https://www.ncbi.nlm.nih.gov/COG) with the BLASTx program (https://www.ncbi.nlm.nih.gov/BLAST/) at an E-value threshold of 1e-5 to evaluate sequence similarity to genes of other species. Gene Ontology (GO) annotation was performed using the Blast2GO software with the Nr annotation results for the unigenes (Conesa et al., 2005). Unigenes with the top 20 highest scores and no shorter than 33 HSP (High-scoring Segment Pair) hits were selected for Blast2GO analysis (Table 1). Then, functional classification of the unigenes was performed using the WEGO software (Ye et al., 2006).

**TABLE 1:** Description of full-length sequencing in *Cipangopaludina chinensis*.

Type	Number	Min length	Average length	Max length	N50
Polymerase	959,578	52	113,116	521,756	175,815
Subreads	26,658,437	51	2,154	274,227	2,466
CCS	818,895	52	2,428	818,895	2,614
FLNC	697,861	50	2,315	15,192	2,530
Unigene	26,312	64	2,572	14,031	2,904

### 2.5 Structure Analysis

Open reading frames (ORFs) were detected using the ANGEL software with the unigene sequences to obtain CDSs, protein sequences, and UTR sequences (Shimizu et al., 2006). Protein-coding sequences of the unigenes were aligned with hmmscan to Animal TFdb (https://bioinfo.life.hust.edu.cn/AnimalTFDB/#!/) to predict TF families. CNCI (version 2) was used to assess the protein-coding potential of transcripts without annotations with default parameters for potential ncRNAs (Sun et al., 2013). ncRNAs were classified according to their secondary structures and sequence conservation.

### 2.6 Simple Sequence Repeats Prediction and Primer Design

The MIcroSAtellite (MISA, https://pgrc.ipk-gatersleben.de/misa/) tool was employed for microsatellite mining across the whole transcriptome. Initially SSRs of 1–6 nucleotide units were identified with the minimum repeat unit number defined as 15 for mono-nucleotides, 6 for di-nucleotides, 5 for tri-nucleotides, and 4 each for tetra-, penta-, and hexa-nucleotides. If the distance between two SSRs was shorter than 100 bp, they were considered one SSR.

### 2.7 Alternative Splicing Detection

To analyze AS events in the transcript unigenes, COding GENome reconstruction Tool (Cogent) was first used to partition transcripts into gene families based on *k*-mer similarity and reconstruct each family into a coding reference genome based on De Bruijn graph methods (Li et al., 2017). Then, the SUPPA tool was used to analyze AS events in the transcript unigenes (Alamancos et al., 2015).

## 3 Results

### 3.1 Single-Molecule Real-Time Sequencing Data Analysis

**FIGURE 1:**
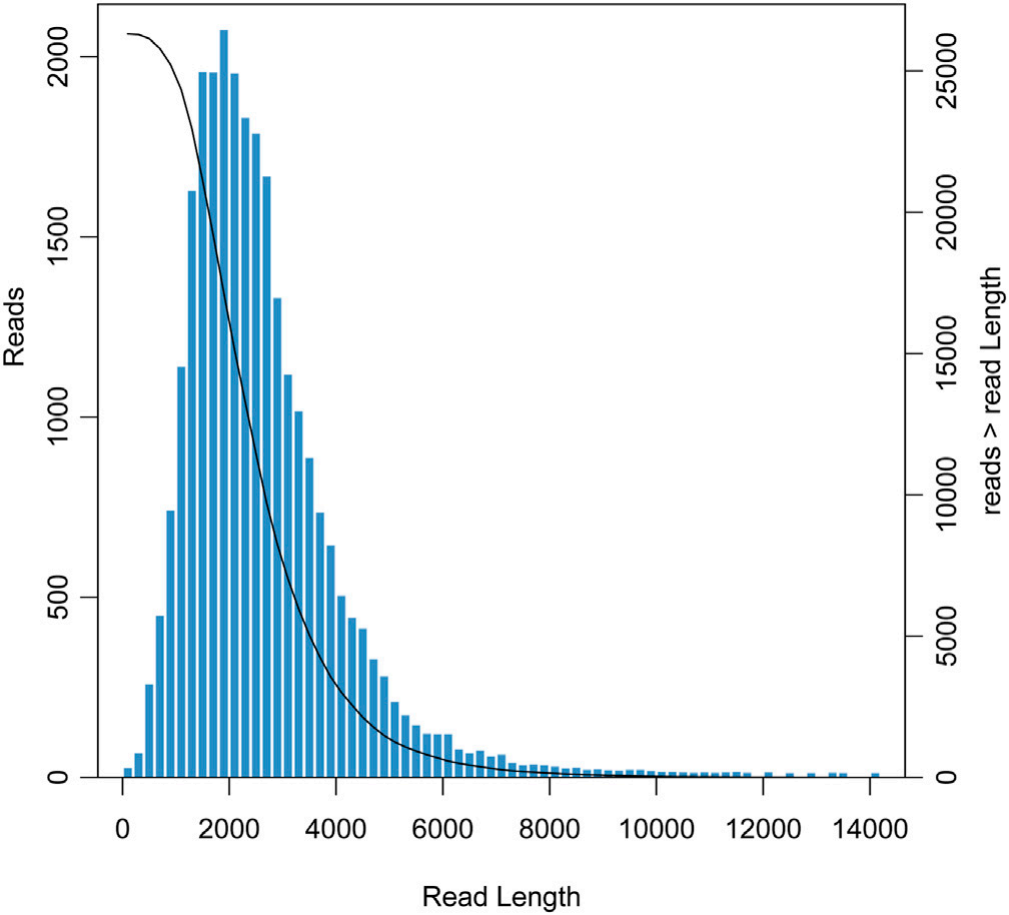
Length distribution of unigenes obtained from the *Cipangopaludina chinensis* library.

Using the PacBio Sequel II sequencing platform, we obtained 57,428,331,313 bp of original transcript data (about 57 Gb) and a total of 26,658,437 subreads with an average subread length of 2,154 bp and an N50 length of 2,466 bp. After CCS analysis, 818,895 CCS reads were obtained with a mean CCS read length and pass number of 2,428 bp and 29, respectively. By clustering and correction, 697,861 FLNC reads were identified with an average length of 2,315 bp and an N50 length of 2,530 bp. Finally, the redundant reads were removed by CD-HIT and 26,312 unigenes with a mean length of 2,572 bp were obtained (Table 1). The length distribution of unigenes is shown in Figure 1. The majority of unigenes were between 1,000 and 5,000 bp, and the longest unigene was about 14,000 bp.

### 3.2 Functional Annotation of *C. chinensis* Sequences

**FIGURE 2:**
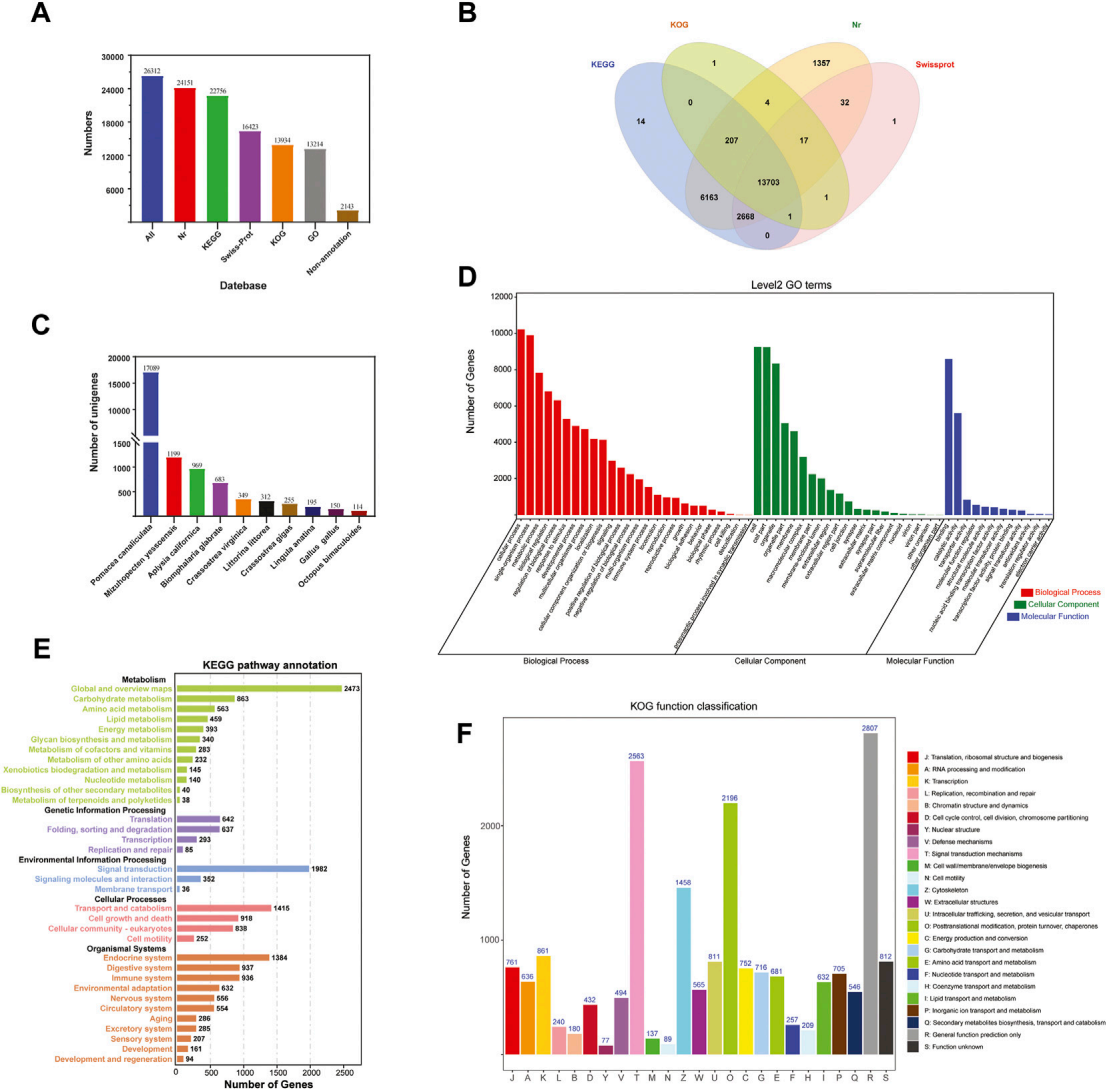
Functional annotation of *Cipangopaludina chinensis*. **(A)** Statistics of the transcripts annotated in different databases. **(B)** Venn diagram of annotations in the NR, GO, KEGG, KOG, and Swiss-Prot databases. **(C)** Distribution of the top 10 species with matched transcripts in the NR database. **(D)** Distribution of GO terms for all annotated transcripts in the biological process, cellular component, and molecular function ontologies. **(E)** KEGG pathways enriched in the transcripts. **(F)** COG categories of the transcripts.

The 26,312 unigenes were annotated using four databases, with 24,151 (91.79%), 22,756 (86.49%), 13,934 (52.96%), and 16,423 (62.42%) unigenes matched to the Nr, KEGG, KOG, and Swiss-Prot databases, respectively (Figure 2A). A total of 24,169 (91,86%) unigenes were annotated in all the databases (Figure 2B). After aligning the unigene sequences with the Nr database using BlastX, the sequence with the best comparison result for each unigene was taken as the corresponding homologous sequence, the species to which the homologous sequence belonged was determined, and the homologous sequences of each species were counted. The results showed that the species with the highest homology was *Pomacea canaliculata* (17,089 unigenes), followed by *Mizuhopecten yessoensis* (1,199) and *Aplysia californica* (969) (Figure 2C).

A total of 13,214 sequences were annotated using the GO function classification library under the three broad categories: molecular function (17,422 unigenes), cellular component (48,494), and biological process (80,752). “Binding” (8,587, 49.29%), “cell” (8,587, 17.71%), and “cellular process” (10,215, 12.65%) were the most common terms among the annotated unigenes in the three categories mentioned above (Figure 2D).

In KEGG pathway analysis, the unigenes were assigned to five main categories: cellular processes (4,595 unigenes), environmental information processing (4,728), genetic information processing (1,797), metabolism (8,156), and organismal systems (10,363). The signal transduction (4,297, 16.33%) category had the largest number of unigenes. Next was infectious diseases (4,230, 16.08%) and cancers (3,068, 11.66%) (Figure 2E). Based on the KEGG annotation information we were able to further obtain Pathway annotations for unigene and focus on some of the more applicable pathways including PI3K-Akt signaling pathway (436, 5.34%), cGMP-PKG signaling pathway (389, 4.77%), Drug metabolism-cytochrome P450 (91, 1.12%), Platinum drug resistance (127, 1.56%), AMPK signaling pathway (267,3.27%), NF-kappa B signaling pathway (119, 1.46%), T cell receptor signaling pathway (71, 0.87%), Toll and Imd signaling pathway (80, 0.98%) (Table 2). From these, we also identified some candidate genes involved in drug metabolism and immune related, such as cytochrome P450 family (CYP1A1, CYP2J and CYP2U1), phosphoinositide-3-kinase regulatory subunit (PIK3), cGMP-inhibited 3′,5′- cyclic phosphodiesterase A (PDE3A), glutathione S-transferase (GST), 5′-AMP-activated protein kinase (PRKAG), NF-kappa-B inhibitor alpha (NFKBIA), cell division control protein 42 (CDC42), baculoviral IAP repeat-containing protein 2/3 (BIRC2_3), and others (Table 2). These provide data for the subsequent development of biologic drugs in snail.

**TABLE 2:** Regional distribution of some SSRs in the full-length transcripts of *Cipangopaludina chinensis*.

Type	Number	Ratio	5UTR	CDS	3UTR
Mono-	688	11.4	72	7	609
Di-	2761	45.73	304	204	2253
Tri-	1035	17.14	205	358	472
Tetra-	512	8.48	47	22	443
Penta-	34	0.56	7	1	26
Hexa-	40	0.66	7	17	16
Complex	967	16.02	43	76	848

All unigenes were classified into 25 categories in the KOG functional classification. (Figure 2F). The highest number of unigenes was annotated to the general function prediction only category (2,807, 10.67%), followed by the signal transduction mechanisms (2,563, 9.74%) and posttranslational modification, protein turnover, and chaperones (2,196, 8.35%) categories.

### 3.3 Structure Analysis of *C. chinensis*

#### 3.3.1 Coding DNA Sequences Prediction

**FIGURE 3:**
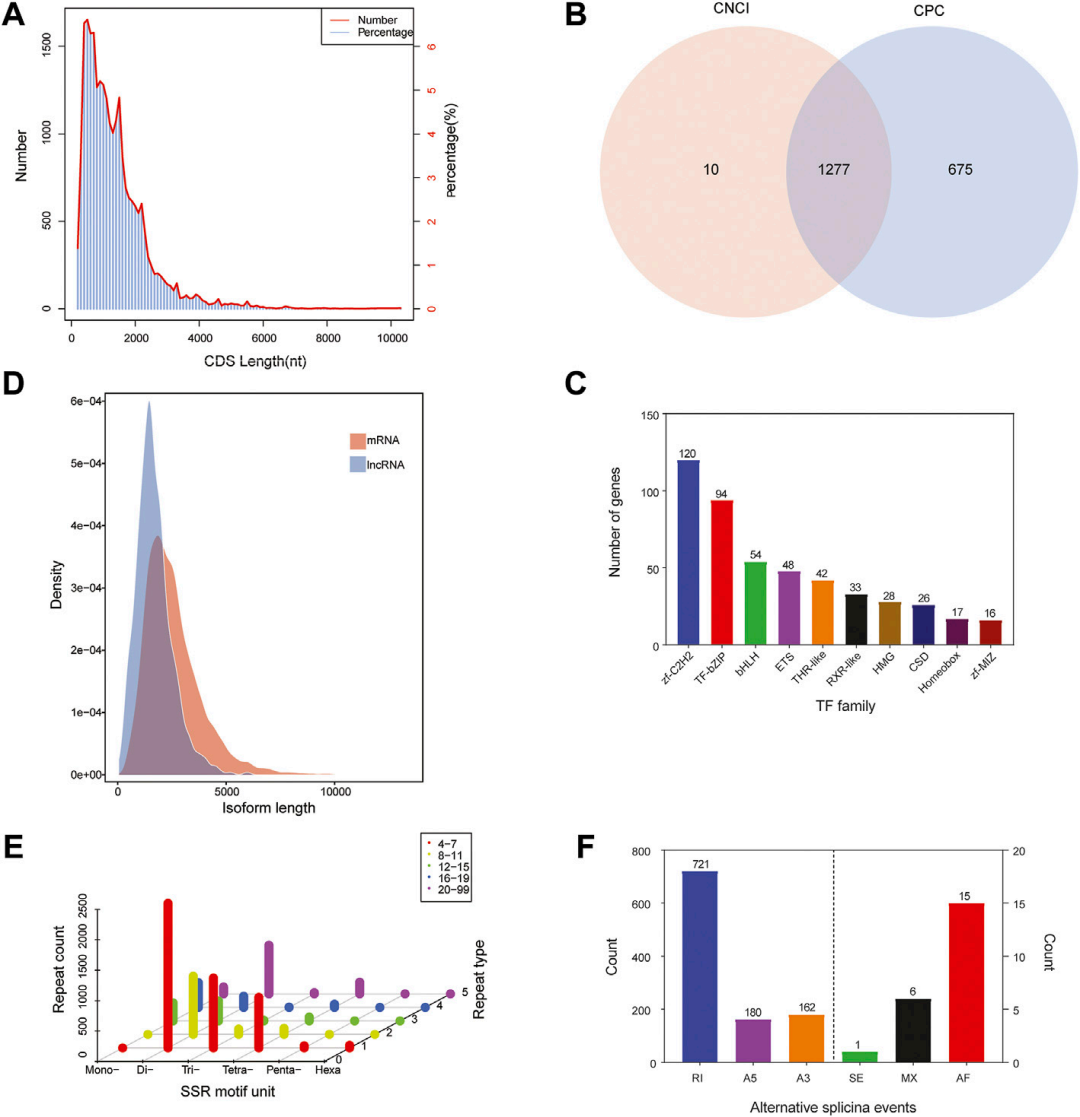
Sequence structure analysis of *Cipangopaludina chinensis*. **(A)** Length distribution of CDSs. **(B)** Venn diagram of lncRNAs identified by the CNCI and CPC methods. **(C)** Length density distribution of the lncRNA and mRNA transcripts. **(D)** Families and numbers of the top 10 TFs produced by SMRT. **(E)** Summary of SSR types in the full-length transcripts of *C. chinensis*. **(F)** Alternative splicing events predicted by SUPPA.

The numbers and lengths of 5′ UTRs, 3′ UTRs, and CDSs were identified using the ANGEL software. 5′ UTRs were observed in 23,559 unigenes and the lengths were mostly less than 1,000 nt, while 3′ UTRs were observed in 23,772 unigenes and the lengths were mainly less than 3,000 nt. We predicted that there were 24,991 CDSs in the library. Most of the CDSs (20,491 unigenes, 81.99%) were less than 2,000 nt in length, while 4,396 unigenes (17.59%) were in the range of 2,000–5,000 nt and only 104 unigenes (representing 0.42%) exceeded 5,000 nt (Figure 3A).

#### 3.3.2 Identification of ncRNAs and Transcription Factors

ncRNA analysis was performed on 2,143 full-length transcribed sequences that were not annotated to the four major databases. We identified 1,287 and 1,952 ncRNAs using CPC and CNCI, respectively (Figure 3B). Integrating the results of these two methods, a total of 1,277 ncRNA transcripts were predicted, accounting for only 4.85% of the total data. Additionally, comparing the length distribution density of mRNAs with the predicted ncRNAs showed that the proportion of ncRNAs in the 64–2,000 nt range was greater than that of the mRNAs (Figure 3C).

TFs are protein molecules with a specific structure and function in regulating gene expression in aquatic animals. We compared the predicted protein sequences with the Animal TFdb database using the hmmscan software and identified 658 TFs from 48 TF families. In Figure 3D, the top 10 TF families are listed, among which zf-c2h2 family (120, 18.24%) has the largest number, followed by the IF-bZIP family (94, 14.29%) (Figure 3D).

#### 3.3.3 Simple Sequence Repeats Analysis

The MISA software was used to search all unigenes of the transcriptome and a total of 8,058 SSRs were found. The SSR statistics showed that the most abundant type was di-nucleotide repeat sequences (4,647), accounting for 57.67%, followed by tri-nucleotide (1,274, 15.81%), tetra-nucleotide (1,239, 15.37%), and mono-nucleotide repeats (816, 10.13%). However, the proportions of penta-nucleotide and hexa-nucleotide repetitive sequences were very small, only 0.48 and 0.53%, respectively. In addition, most SSRs had 4–7 repeat units (Figure 3E). Because CDSs were not predicted for some transcripts, the total count of SSRs in different regions was 6,037. Among these regions, 3′ UTRs had the most (4,667), followed by 5′ UTRs and CDSs with the same number, 685 (Table 2).

#### 3.3.4 Alternative Splicing Prediction

Based on the Cogent software assembly sequence as a reference, the SUPPA software was used for AS event prediction. In our study, a total of 1,085 unigenes were assigned to six AS events (alternative 3′ splice sites, alternative 5′ splice sites, alternative first exons, mutually exclusive exons, retained introns, and exon skipping). Retained introns (RIs) accounted for the highest proportion of all events, at 66.45% (721). Next were alternative 3′ splice sites (A3) and alternative 5′ splice sites (A5), with 180 (16.59%) and 162 (14.93%) events, respectively. The other three events, alternative first exons (15, 1.39%), mutually exclusive exons (6, 0.55%), and exon skipping (1, 0.09%), accounted for only 2.03% (Figure 3F).

## Discussion

SMRT sequencing has the characteristics of high accuracy, reliability, long reading, and sequencing speed, and it enables animal genomic research without a reference genome (Hestand & Ameur, 2019; Jayakumar & Sakakibara, 2019; Zhang et al., 2019; Xiao & Zhou, 2020; Deng et al., 2021). Since the *C. chinensis* is an economically important snail with high medicinal value, and it has vital scientific research potential (Zhou et al., 2022). The current study was the first to obtain full-length transcriptome data of the snail using third-generation sequencing technology. By data analysis, 26,312 unigenes with a mean length of 2,572 bp were obtained in the study. Deng et al. used the same technology to sequence the no-reference-genome species *Coelomactra antiquata* and found a total of 39,209 unigenes with an average length of 2,732 bp (Deng et al., 2021). Similarly, Yang et al. found 63,801 full-length transcripts in the three developmental stages of *Rhynchophorus ferrugineus*, with an average length of 2,964 bp (Yang et al., 2020). Compared with the above organisms, the transcript data volume and sequence length of *C. chinensis* were relatively small.

We performed functional annotations on all the obtained transcripts in the four major databases, of which 91.86% of unigenes were successfully annotated and only 8.14% were unannotated. The percentage of annotated unigenes is very high, possibly because there have been many molecular biology studies on gastropod mollusks (e.g., *P. canaliculata, Cipangopaludina cathayensis*, and *Tegillarca granosa*) in the past, and the data collected in these databases are relatively complete; thus, the genomic information of *C. chinensis* can be compared with existing data (Chen et al., 2017). According to the species distribution statistics of the NR database, *P. canaliculata* has the most homologous sequences, revealing that *P. canaliculata* is closely related to *C. chinensis*. This coincides with the findings of Mu et al., who performed an evolutionary relationship analysis based on the mitochondrial genome, which suggested that *P. canaliculata* has a close evolutionary relationship with *C. cathayensis* (homologous to *C. chinensis*) (Mu et al., 2015). In terms of the annotation results from the GO, KEGG, and KOG databases, the number of transcripts identified in KEGG was highest, among which the numbers of transcripts involved in intracellular signal transduction, infectious diseases, and cancer were the largest. In-depth analysis also observed a number of pathways and candidate genes involved in drug metabolism and immune response (Table 2). Previously, some scholars found that *C. chinensis* flesh is rich in biopolysaccharides and confirmed that they play an active role in anti-tumor activity, lowering blood sugar, and anti-hepatitis B virus activity (Jiang et al., 2013; Shi et al., 2016; Xiong et al., 2020). This implies that *C. chinensis* genes may have many biological activities, participate in material metabolism and immune processes in animals, and have important biomedical value.

The structure of the non-redundant unigenes was also analyzed in our study. A total of 1,277 ncRNAs and 8,058 SSRs were identified. ncRNAs have complex and precise regulatory function in cell differentiation, ontogeny, and gene expression (Wu et al., 2014; Quan et al., 2018). The transcripts produced by 4–9% of mammalian genome sequences are ncRNAs (Lu and Huang, 2017; Han et al., 2018). In recent years, research on ncRNAs has progressed rapidly, but the functions of most ncRNAs are still unclear, especially in lower animals (Niu et al., 2019). In contrast, research on SSRs has been relatively comprehensive and SSRs have been widely used in practical applications, such as genetic diversity analysis, genetic map construction, and molecular marker development (Ali et al., 2019; Sun et al., 2019; Cregan et al., 2020) It was reported that 16,717 and 20,106 SSRs were found in *P. canaliculata* and *C. antiquata*, respectively, based on transcriptome sequencing (Mu et al., 2015). This shows that the number of SSRs found in *C. chinensis* in this study was relatively low. Furthermore, we also predicted the TFs of *C. chinensis* and obtained detailed TF numbers and family classifications, among which the zf-c2h2 family was the most abundant. In plants, zf-c2h2 TFs participate in all aspects of growth and development (especially root and floral development), as well as biotic and abiotic stress responses (Agarwal et al., 2007; Jiang & Pan, 2012; Liu et al., 2015). In animals, zf-c2h2 TFs are more involved in tumors, cancers, and related gene regulation (Hu et al., 2019; Hu et al., 2021). However, the role of zf-c2h2 TFs in the lower animal *C. chinensis* needs further research.

## Conclusion

*C. chinensis* is an aquatic animal product with high economic value and has potential for medicinal development. More importantly, it plays an important role as a carbon sink in the ecosystem. In this study, PacBio SMRT sequencing technology was used to successfully construct a high-quality transcript library of *C. chinensis* (26,312 unigenes with an average length of 2,572 bp), and the sequences were functionally annotated (including 24,151 Nr, 22,756 KEGG, 13,934 KOG, and 16,423 Swiss-Prot sequences) and structurally analyzed (including 24,991 CDSs, 1,277 ncRNAs, 6 AS events, 658 TFs, and 8,058 SSRs) using bioinformatics software. These results provide comprehensive scientific data for future research on functional gene mining, molecular breeding, and biomedicine research of *C. chinensis.*

## Data Availability

The original contributions presented in the study are publicly available. This data can be found here: https://www.ncbi.nlm.nih.gov/bioproject/PRJNA817831/.
